# Feasibility study using longitudinal bioelectrical impedance analysis to evaluate body water status during fluid resuscitation in a swine sepsis model

**DOI:** 10.1186/s40635-022-00480-5

**Published:** 2022-12-06

**Authors:** Hwain Jeong, Inwon Park, Jae Hyuk Lee, Dongsung Kim, Sumin Baek, Seonghye Kim, You Hwan Jo

**Affiliations:** 1 Department of Emergency Medicine, Seoul National University Bundang Hospital, 82, Gumi-Ro 173 Beon-Gil, Bundang-Gu, Seongnam-Si, Gyeonggi-Do 13620 Republic of Korea; 2 Department of Emergency Medicine, Seoul National University College of Medicine, 103 Daehak-Ro, Jongno-Gu, Seoul, 03080 Republic of Korea

**Keywords:** Sepsis, Electric impedance, Body fluid compartments, Fluid therapy, Fluid shifts

## Abstract

Fluid resuscitation is crucial in the initial management of sepsis; however, little is known about the serial changes and overall distribution of fluids administered into the body. To identify the feasibility of longitudinal bioelectrical impedance analysis during fluid treatment, a preclinical porcine model of *Escherichia coli-*induced sepsis was used. After sepsis induction, pigs were treated with fluid and vasopressors and monitored for up to 12 h after bacterial infusion or until death. Bipolar electrodes for bioelectrical impedance analysis were attached to the left extremities and measurements were performed every 10 min. Among the 12 subjects, 7 pigs expired during the experiment, and the median survival was 9.5 h. As sepsis progressed with an increase in cumulative fluid balance, R_0_ [∝ 1/extracellular water (ECW)] decreased, while *R*_i_ [∝ 1/intracellular water (ICW)] and ratio of extracellular water to total body water (ECW/TBW) increased. The phase angle constantly decreased throughout the monitoring period, and all non-survivors died when the phase angle decreased by more than 10%. Among the variables, Δ*R*_0_ and Δphase angle showed moderate negative correlations, and ΔECW/TBW showed a moderate positive correlation with the hourly fluid balance. Compared to survivors, a greater increase in ΔECW/TBW and a decrease in phase angle were observed in non-survivors over time, with an increase in cumulative fluid balance. Differences in ΔECW/TBW and phase angle emerged at 240 min when the difference in cumulative fluid balance between the two groups (survivors vs non-survivors) exceeded 1000 mL. In conclusion, continuous measurements of bioelectrical impedance analysis in a porcine sepsis model are feasible and may reflect changes in the body water profile during fluid resuscitation.

**Supplementary Information:**

The online version contains supplementary material available at 10.1186/s40635-022-00480-5.

## Introduction

Fluid resuscitation is crucial in the initial management of sepsis because it helps improve tissue hypoperfusion [1]. However, excessive hydration may be associated with adverse effects such as pulmonary edema, leading to a longer duration of organ support, longer ICU or hospital stay, and increased mortality [2–4]. These complications can be much more lethal to special populations with renal impairment or cardiac dysfunction [2, 5, 6]. Therefore, accurate patient monitoring during fluid treatment and the consequent titration of adequate fluid volume are of paramount importance [7].

Moreover, little is known about the serial changes and overall distribution of fluids administered into the body [5]. As distributive shock is the mainstream pathophysiology of sepsis due to systemic cytokine storm and increased vascular permeability, only a portion of administered fluids turn into intravascular volume [8–10]. Therefore, assessing the consequent water distribution would help in determining an adequate volume for administration to restore the effective volume [11, 12].

Bioelectrical impedance analysis (BIA) has recently been proposed as a useful tool for assessing the volume status. It is noninvasive, inexpensive, and can provide objective real-time measurements [5, 13, 14]. Multiple studies have shown that BIA demonstrates good correlations with body water measurements [5, 15]. Furthermore, the proportion of extracellular and intracellular water can also be calculated using the differences in measured variables [13–16].

Therefore, to verify the feasibility of BIA for continuously monitoring real-time changes in body water distribution during fluid resuscitation in sepsis, a preclinical controlled study was conducted. To implement an ideal experimental sepsis model with fluid treatment, pigs with anatomic, physiologic, and hemodynamic features similar to humans were used [17–19]. This study aimed to investigate the longitudinal, real-time changes in BIA measurements during fluid resuscitation, specifically between survivors and non-survivors, in the first 12 h of sepsis.

## Materials and methods

### Study settings

All experiments were approved by the Institutional Animal Care and Use Committee of Seoul National University Bundang Hospital (protocol no. BA-2104-317-029–03), and animals were maintained in the facility accredited by AAALAC International (#001847) in accordance with the Guide for the Care and Use of Laboratory Animals.

### Animal preparation

Twelve male three-way crossbred (Yorkshire Berkshire Duroc, Cronex, Seoul, Republic of Korea) pigs (46.0 kg, [44.3–49.3]) were used as study subjects. Prior to the experiment, the pigs were housed in a controlled animal facility with adequate access to food and water. Anesthesia was induced by intramuscular injection of zolazepam/tiletamine (Zoletil, 5 mg/kg; Virbac, Carros, France) and xylazine (Rompun, 5 mg/kg; Elanco, Greenfield, IN, USA). After anesthesia administration, the bilateral neck and inguinal areas were shaved, followed by scrubbing with a povidone-iodine soap. To monitor vital signs, a pulse oximeter, 3-lead electrocardiography monitor, and rectal thermometer were used (IntelliVue, Patient Monitor MP20; Philips, Amsterdam, Netherlands). The pigs were intubated using a 7-Fr endotracheal tube connected to a mechanical ventilator (Drager Fabius GS, Lubeck, Germany). General anesthesia was maintained via inhalation of 2–3% sevoflurane (Baxter Inc., Deerfield, IL, USA). Volume-controlled ventilation was initially applied with the following settings: tidal volume, 6 mL/kg; positive end-expiratory pressure, 5 cmH_2_O; fraction of inspired oxygen, 0.21; respiratory rate, 12–15/min. The components were regulated to maintain an arterial PaO_2_ ≥ 65 mmHg and a PCO_2_ of 40–45 mmHg, as measured by arterial blood gas analysis.

After thorough preoperative dressing with povidone-iodine, surgical draping was performed on the abdomen, from both axillae to the inguinal region. Under ultrasonographic guidance, 6-Fr arterial catheters (Merit Medical, South Jordan, UT, USA) were placed in both the femoral arteries. Each catheter was used for repetitive blood sampling and for continuous invasive blood pressure monitoring. A Swan–Ganz catheter (Model 131HF, 7 Fr; Edwards Lifesciences, Irvine, CA, USA) was inserted into the right external jugular vein for monitoring blood temperature, cardiac output, and pulmonary capillary wedge pressure (PCWP). A central venous catheter (ARROW CVC; Teleflex, Morrisville, NC, USA) was inserted into the left external jugular vein and was used for fluid and vasopressor administration. A Foley’s catheter was placed via suprapubic cystostomy to measure the precise urine output.

### Bioelectrical impedance analysis

InBody M20 (Inbody Co., Seoul, Republic of Korea) was used to evaluate body water status. Two bipolar electrodes, which were applied to the left hand and left foot, were connected to the InBody M20. InBody M20 measured four components: impedance (Z), resistance (*R*), reactance (Xc), and phase angle (θ), using three different frequencies (5 kHz, 50 kHz, and 250 kHz). Briefly, when an electrical current is transmitted through the body, tissues exhibit various degrees of resistance (*R*) which is the opposition to the flow of electrical current. Reactance (Xc) is the opposition to a current change due to the capacitance of the human body composition. Impedance (Z) is the vector analysis of resistance (*R*) and reactance (Xc). During the passage of electrical current through the cell membrane, reactance (Xc) causes a time delay, creating a phase shift between voltage and current that extracts the phase angle (θ). The detailed explanation of variables is presented elsewhere [16, 20].

ECW/TBW, the ratio of extracellular water to total body water, was measured using the built-in BIA software [21]. Longitudinal measurements were performed every 10 min during the experiment.

*R*_0_, *R*_inf_, and *R*_i_ were acquired from the values measured in the bioelectrical impedance analysis [21]. The resistance *R*_0_ is inversely proportional to ECW, the resistance *R*_i_ is inversely proportional to ICW, and is calculated from resistance *R*_0_ and *R*_inf_. To evaluate the resistance over time, the ratio of change in resistance [Δ*R*_0_ (%), Δ*R*_inf_ (%), Δ*R*_i_ (%)] at each time point was calculated as follows:

$$\Delta R\mathrm{x }\left(\mathrm{\%}\right)=\frac{R\mathrm{\, at \,the \,point}-R\mathrm{\, at \,the \,baseline}}{R\mathrm{\, at \,the \,baseline}} \times 100.$$

To identify the correlation between the hourly fluid balance and change in variables [Δ*R*_0_ (Ω), Δ*R*_inf_ (Ω), Δ*R*_i_ (Ω), ΔECW/TBW, and Δphase angle (°)], the variables 1 h before were subtracted from the variable at the point. In the comparison between survivor and non-survivor analyses, ΔECW/TBW was calculated by subtracting the variables at the baseline from the variables at the point.

### Induction of the swine bacteremia model

To induce sepsis in pigs, we used extended-spectrum β-lactamase (ESBL)-producing *Escherichia coli* (*E. coli*; 5.0 × 10^9^ CFU, Strain: BAA-196, ATCC, Manassas, VA, USA). After preparing the pigs as previously described, the diluent of ESBL-producing *E. coli* in 1000 mL of normal saline was administered intravenously over 1 h. To generate the survivor and non-survivor groups before the end of the experiment (12 h) based on our previous modeling experience [22, 23], ceftriaxone (2 g, Cerixone, Chong Kun Dang, Seoul, Republic of Korea) was administered 1 h after completion of bacterial infusion.

Pigs were monitored for up to 12 h after bacterial infusion or until death. The maximal support described below was provided to maintain a mean arterial pressure (MAP) > 65 mmHg. The maintenance fluid (plasma solution A, HK inno. N; Seoul, Republic of Korea) was administered at a rate of 2 mL/kg/h. When the MAP dropped below 65 mmHg, fluid challenge of 30 mL/kg was performed with Plasma Solution A, and when it was insufficient, vasopressors were added in the order of norepinephrine, vasopressin, and epinephrine. Additional intermittent fluid loading was attempted based on the response of MAP and increase in vasopressor dose was preferred over fluid loading when the response was unidentified.

### Measurements and calculations

The following variables were continuously monitored and recorded every 30 min: invasive arterial blood pressure, heart rate (HR), rectal and blood temperature, lead II electrocardiographic variables, pulse oximetry variables, pulmonary arterial pressure (PAP), central venous pressure (CVP), pulmonary capillary wedge pressure (PCWP), fluid input, and urine output.

Cardiac output (CO) was measured three times every 1 h using the thermodilution technique via the Swan–Ganz catheter (Edwards 111F7P; Edwards Lifesciences Corp., Irvine, CA, USA) and the median values are presented. Stroke volume (SV) was calculated with cardiac output and heart rate (SV = CO/HR). Systemic vascular resistance (SVR) was calculated with MAP, CVP, and cardiac output (SVR (dyn/s/cm^−5^) = (MAP − CVP) $\times$ 80/CO). The following laboratory tests were conducted every 1 h: arterial blood gas analysis (ABGA), including the P/F ratio (ratio of partial pressure of oxygen to the fraction of inspired oxygen), electrolyte levels, lactate levels (Nova CCX; Nova, Waltham, MA, USA), complete blood cell count (Hemavet 950FS; Drew Scientific Inc., Miami Lakes, FL, USA), and chemistry panel (VetScan; Abaxis, Union City, CA, USA). The sequential organ failure assessment (SOFA) score without the central nervous system (CNS) score was calculated based on the variables described above.

### Statistical analysis

Variables are described as the mean and standard error of the mean. Pearson’s correlation analysis was performed to measure the association between variables. Two-way analysis of variance (ANOVA) for repeated measures with Sidak’s multiple comparisons test (two-way repeated measures ANOVA) was performed to compare the trends in variables between the two groups. *p*-values < 0.05 were considered statistically significant. Statistical analyses and graph generation were performed using Prism 9.2 (GraphPad Software, Inc., San Diego, CA, USA).

## Results

**Fig. 1:**
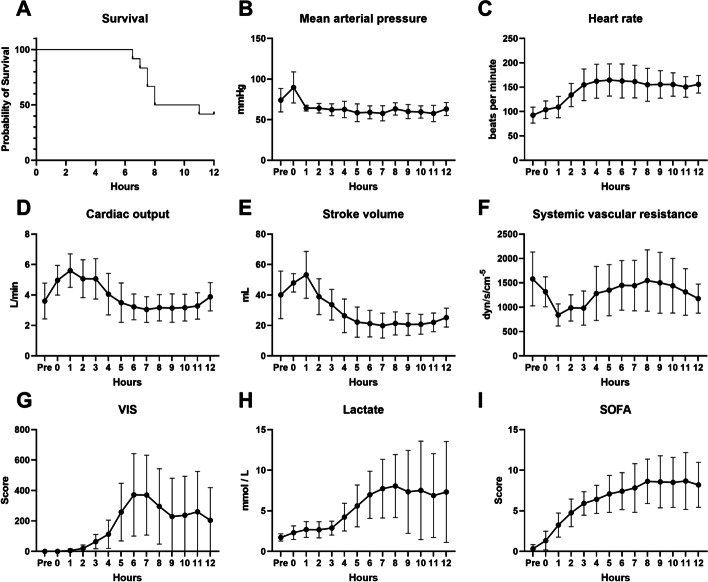
Trends of survival (**A**), hemodynamic variables (**B**–**F**), VIS (**G**), lactate levels (**H**), and SOFA scores (**I**) of 12 pigs in the *E. coli*-induced porcine bacteremia model. Data are presented as the means and standard error of the mean. *VIS* vasopressor-inotrope score, *SOFA* Sequential Organ Failure Assessment

A total of 12 pigs were monitored for up to 12 h after ESBL-producing *E. coli* infusion or until death. Seven animals died during the experiment, resulting in a mortality rate of 58.3% and median survival time of 9.5 h (Fig. 1A).

After bacterial infusion, the MAP was maintained at > 65 mmHg, with maximal fluid and vasopressor support (Fig. 1B). Heart rate showed a tendency to increase for the first 4 h and was maintained at approximately 150 beats per minute thereafter (Fig. 1C). Cardiac output and stroke volume increased with a decrease in systemic vascular resistance until 1 h after completion of bacterial injection, followed by the opposite trend thereafter (Fig. 1D–F). The lactate level, vasopressor-inotrope score (VIS), and SOFA score (excluding the CNS score) increased until 7 or 8 h, and the median value started decreasing due to the decrease in deceased subjects (Fig. 1G–I, Additional file 1: Fig. S1).

**Fig. 2:**
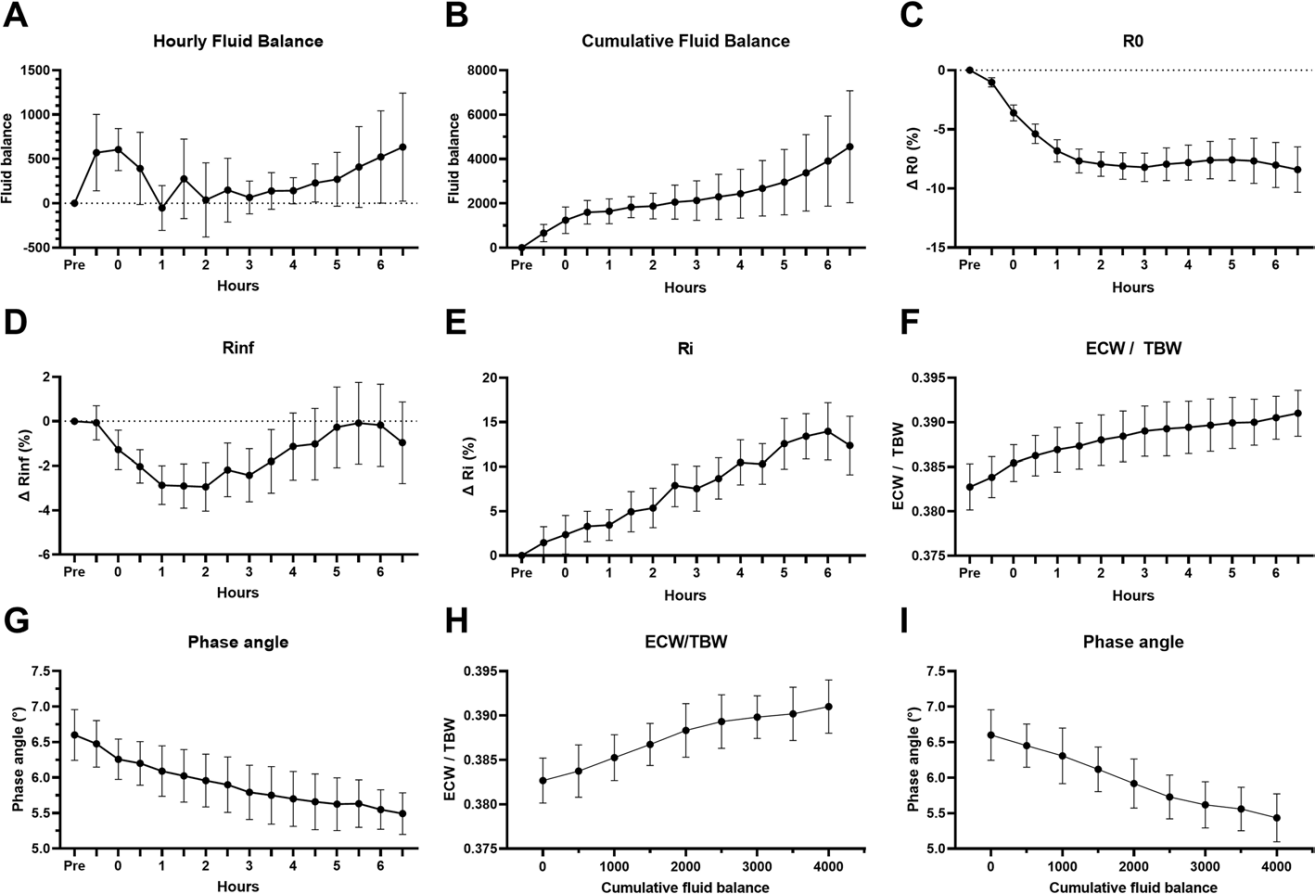
Trends of fluid balances (**A**, **B**) and measured bioelectrical impedance analysis variables (**C**–**G**) over time, and trends of ECW/TBW (**H**) and phase angle (**I**) over cumulative fluid balance of 12 pigs in the *E. coli*-induced porcine bacteremia model. Data are presented as the means and standard error of the mean. *ECW* extracellular water, *TBW* total body water

The hourly fluid balance (Fig. 2A) and cumulative fluid balance (Fig. 2B) increased continuously during the experiment. The resistance *R*_0_ (∝ 1/ECW) decreased, and correspondingly, the resistance *R*_i_ (∝ 1/ICW) increased (Fig. 2C–E). The ratio of ECW to TBW (ECW/TBW) and phase angle continued to increase and decrease, respectively, over time (Fig. 2F and G). Both variables showed the same trend over the cumulative fluid balance (Fig. 2H and I).

**Fig. 3:**
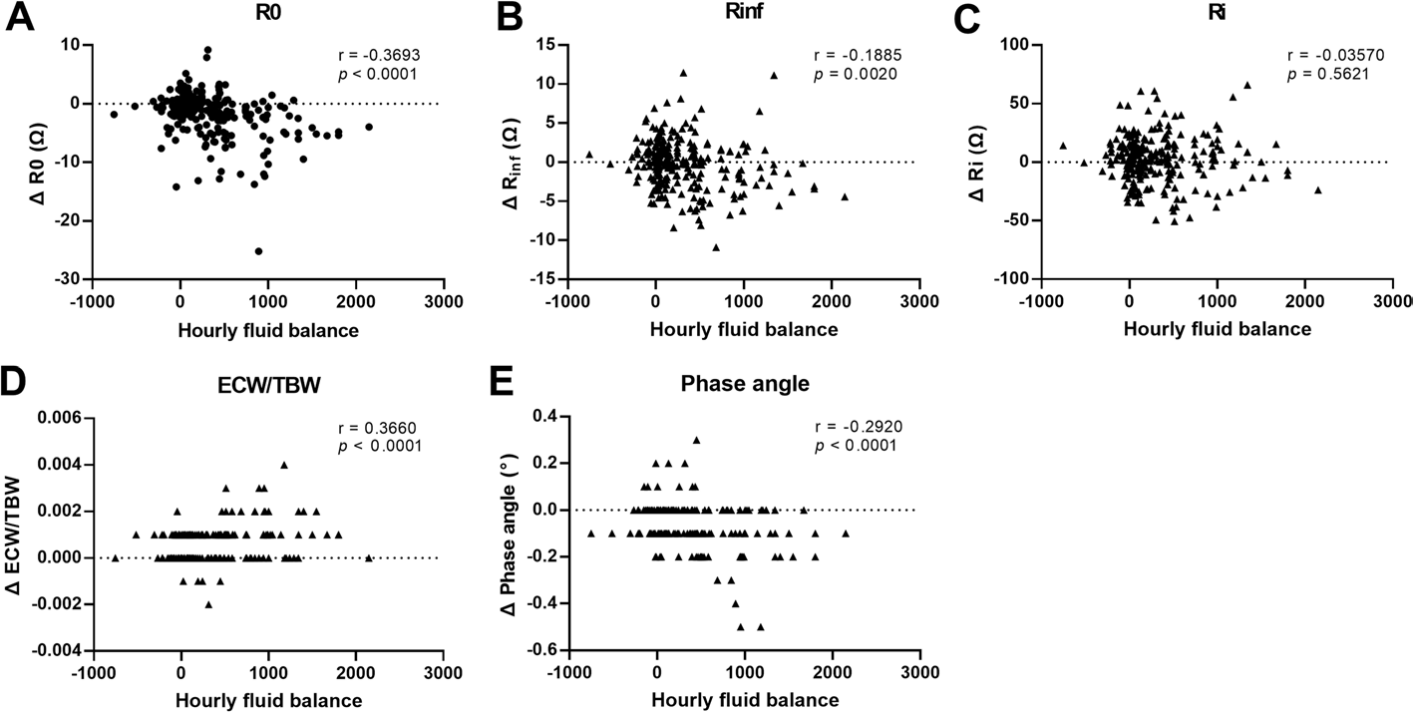
Pearson’s correlation analyses between changes of resistances (ΔR_0_(**A**), ΔR_inf_(**B**), and ΔR_i_(**C**)), ECW/TBW (**D**), and phase angle (**E**) with fluid balance. Correlation coefficient *r* values with *p* values are denoted in each graph. *ECW* extracellular water, *TBW* total body water

Changes in the resistance (Δ*R*_0_, Δ*R*_inf, and_ Δ*R*_i_), ECW/TBW (ΔECW/TBW), and phase angle (Δphase angle) at each hour were measured to evaluate the correlation with the hourly fluid balance. While Δ*R*_i_ (*r* = − 0.0357, *p* = 0.5621) showed no significant relationship, ΔR_0_ (*r* = − 0.3693, *p* < 0.0001) and Δ*R*_inf_ (*r* = − 0.1885, *p* = 0.0020) showed moderate and mild negative correlations, respectively, with fluid balance (Fig. 3A–C). ΔECW/TBW (*r* = 0.3660, *p* < 0.0001) and Δphase angle (*r* = − 0.2920, *p* < 0.0001) showed moderate positive and negative correlations with hourly fluid balance, respectively (Fig. 3D and E).

**Fig. 4:**
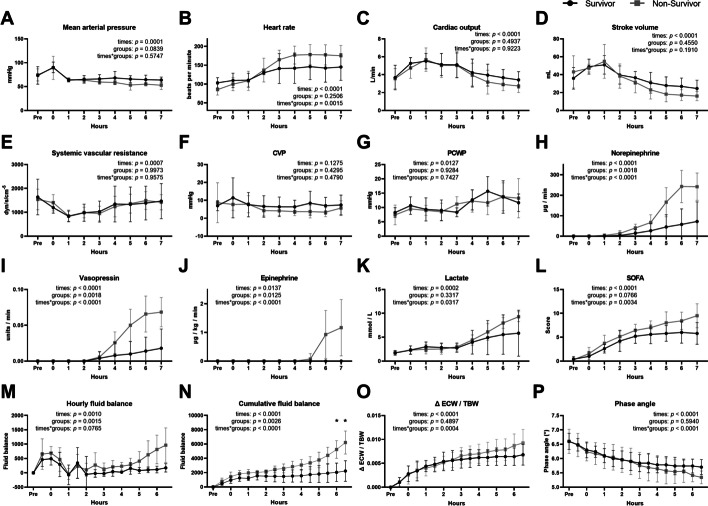
Comparisons of trends in hemodynamic variables (**A**–**F**), PCWP (**G**), vasopressors (**H**–**J**), lactate (**K**), SOFA score (**L**), hourly fluid balance (**M**), cumulative fluid balance (**N**), ΔECW/TBW (**O**), and phase angle (**P**) between survivors (*n* = 5) and non-survivors (*n* = 7) in the *E. coli*-induced porcine bacteremia model. Data are presented as the means and standard error of the mean. The *p* values between times, groups, and times*groups using two-way RM ANOVA are denoted in each graph. Asterisks (*) indicate statistical significance of difference (*p* < 0.05) using Sidak’s multiple comparison tests. *CVP* central venous pressure, *PCWP* pulmonary capillary wedge pressure, *SOFA* sequential organ failure assessment, *ECW* extracellular water, *TBW* total body water

Next, the difference in the trend of variables between survivors and non-survivors was evaluated (Fig. 4). Compared to survivors, increase in HR with decrease in SV was prominent in non-survivors after 2 h, whereas CO was similar between the two groups (Fig. 4A–D). Then, from 4 h, CO started to be lower in the non-survivor group while SVR showed no significant difference between 2 groups (Fig. 4E–G). The dose of vasopressors, lactate, and SOFA (excluding CNS score) score showed greater increase in non-survivors (Fig. 4H–L). The hourly and cumulative fluid balance tended to continuously increase over time, with a greater increase in non-survivors (Fig. 4M and N). The trend of cumulative fluid balance over time was significantly different between the two groups (*p* < 0.0001 between times, *p* = 0.0026 between groups, and *p* < 0.0001 between times × groups; two-way repeated measures ANOVA), which resulted in significant differences at 6 and 6.5 h (*p* = 0.0357 and *p* = 0.0210; Sidak’s multiple comparison tests). ΔECW/TBW increased in both groups, and the trend of ΔECW/TBW was significantly different between survivors and non-survivors, with a greater increase in non-survivors (Fig. 4O, *p* < 0.0001 between times, *p* = 0.4897 between groups, and *p* = 0.0004 between times*groups). The phase angle decreased in both groups, and the trend of the phase angle was significantly different between survivors and non-survivors, with a greater decrease in non-survivors (Fig. 4P, *p* < 0.001 between times, *p* = 0.5940 between groups, and *p* < 0.0001 between times × groups). The time period in which the distinction became evident in ΔECW/TBW and phase angle was 240 min when the difference in the cumulative fluid balance between the two groups started exceeding 1000 mL (Fig. 4N–P).

## Discussion

In this study, we identified that the trend of body water status, including its distribution, can be assessed using BIA. As our experimental sepsis model progressed with a positive cumulative fluid balance derived from intentional fluid therapy, resistances *R*_0_ (∝ 1/ECW) and *R*_i_ (∝ 1/ICW) measured by BIA decreased and increased, respectively. The ratio ECW/TBW exhibited an increasing pattern during the experiment and a positive correlation with the fluid balance. ΔR_0_ showed a moderate negative correlation with fluid balance, indicating that an increase in fluid balance, including ECW, could be assessed with alteration in R_0_. These findings were consistent with the alleged pathophysiology of sepsis involving intracellular dehydration with an increase in extracellular water [24]. In addition, increased ECW/TBW indicates increased water content in the extracellular space, suggesting that the injected fluid did not increase intracellular water. Therefore, BIA can be considered effective for real-time observation of changes in body fluid composition during fluid therapy in patients with sepsis.

During an hour of bacterial infusion (Pre ~ 0), MAP, SV, and CO increased due to increased venous return and preload attributed from the administration of 1000 mL fluid of bacteria diluent. An hour after bacterial infusion (0 ~ 1 h), MAP and SVR decreased while SV increased, suggesting reduced preload and afterload. This might have derived from leftwards movement on the Frank–Starling curve from the overloaded preload status which was induced by the previous infusion of bacteria-diluent fluid. In addition, vasodilation presumed from decreased afterload may have developed during this period due to the endothelial dysfunction including dysregulation of vasoactive molecules (nitric oxide, cytokines) and thereby relaxation of smooth muscle. However, since a massive amount of *E. coli* (5.0 × 10^9^ CFU) should be administered into the blood in a relatively short period for sepsis modeling, infusion of a large volume (1000 mL) of diluent fluid was practically unavoidable. Two hours after bacterial infusion, SV and CO progressively decreased with increase of HR and SVR until the end of the experiment while the MAP was maintained around 65 mmHg. In addition to vasodilation, ventricular dysfunction is also presumed in this stage which decreased contractility during systole (decreased calcium trafficking) and abnormal cardiac filling (decreased calcium flux) during diastole is induced by endotoxin, cytokines, and nitric oxide [25–27]. Although intravascular volume is presumed to be increased as initial bolus and maintenance fluid was provided to the subject, afterload and MAP remained low due to vasodilation with reduced CO and SV. In terms of decreased SV and CO, tissue perfusion was low, as evidenced by a gradual decrease in urine output and deterioration of other SOFA scores. Regarding hemodynamics, the model in this experiment suggests that the administered fluid and vasopressors generated only adverse effects without signs of recovery, consistent with the fatal course of sepsis.

In the comparative analysis of survivors and non-survivors, SV decreased in the non-survivor group who received higher volume of fluid, suggesting that venous return might be reduced despite hypervolemia. Capillary leakage of intravascular volume including the fluid administered due to increased vascular permeability during the experiment is suspected [28, 29]. Additionally, a supraphysiologic increase in HR may also reduce ventricular filling time, thereby decreasing preload [30].

BIA has a strong advantage in that it can be measured in real-time with less time consumption, is noninvasive, and does not require a specific clinical setting such as sedation or mechanical ventilation [16, 31]. Owing to this convenience, several studies using BIA guides in fluid management have been previously reported [20]. A randomized controlled trial of bioimpedance-guided fluid management in peritoneal dialysis demonstrated improvement in fluid overload status, but failed to show the benefit of 1-year survival [32]. Although there are differences in the follow-up period, main outcome, and target population, both studies suggest that an increase in ECW/TBW correlates with volume overload, which may result in detrimental outcomes. In an observational clinical trial of bioelectrical impedance vector analysis (BIVA) in critically ill patients, directional changes in BIVA hydration corresponded with changes in cumulative fluid balance [33]. However, only large amounts of fluid loss (> 2 L) reached statistical significance, suggesting that BIVA is insensitive to small alterations in fluid balance. In the present study, R_0_ was inversely correlated with fluid balance, which was less than 2 L. In a previous clinical study, an increasing trend in ECW/TBW among non-survivors of sepsis during initial fluid resuscitation was identified [8]. However, the study was confined to a small population with various patient characteristics, even though BIA measurements broadly differ by height, weight, sex, age, and ethnicity [8, 15, 34]. Moreover, the study was designed to measure variables before and after fluid resuscitation, which limits our understanding of changes during fluid treatment. In contrast to previous studies, real-time changes in the BIA variables during fluid treatment were identified in this study.

The phase angle, considered an indicator of cell membrane integrity, was also evaluated in this study. In previous studies, the phase angle was found to be lower in non-survivors of sepsis or intensive care surgery than in survivors [14, 35]. Consistent with previous results, the measurement of the phase angle of all the experimental subjects in this study started from a healthy state and gradually decreased with the progression of sepsis. The phase angle of non-survivors showed a steeper decrease than that of survivors, and subjects with a decrease of > 10% from baseline expired during that period. Interestingly, in a previous clinical study of fluid resuscitation on patients with sepsis, the trend of the phase angle decreased but failed to show a significant difference between survivors and non-survivors [8]. This can be explained by the finding that every subject in our experimental study was initiated from a healthy status with an equivalent phase angle, whereas the clinical study showed significant differences from the baseline phase angle, and the premorbid phase angle measurement in patients was not feasible. Moreover, a previous clinical study of critically ill patients reported that changes in the phase angle were inversely related to changes in cumulative fluid balance, which coincides with the results of this study [36]. In this study, ECW/TBW and phase angle may provide comparable data that the size of the intracellular space decreases with increasing ECW, resulting in a smaller phase angle.

In our experimental sepsis study, *R*_0_, *R*_inf_, *R*_i_, and ECW/TBW variables were continuously measured, providing the scope of understanding how the above variables change from a healthy baseline status to the progression of sepsis, and further to the treatment phase. In addition, most clinical studies regarding BIA confront individual variability issues acquired from values influenced by sex, age, and height [37, 38]. Therefore, to minimize confounding factors and consequent interpretations, longitudinal observations in the same disease modeling and treatment are required. In this context, this study can be highlighted with the adoption of a single bacteremia model with an identical fluid and vasopressor treatment strategy that decreases confounding factors derived from disease-category issues or treatment bias. In addition, most previous BIA studies in fluid management were designed to measure the values at intervals of 1 or 2 days, whereas this study focused on real-time monitoring of BIA variables during fluid treatment to assess the correlation between variables and fluid balance [35, 36]. Based on previous clinical studies [8, 37] including the present study, further clinical trials are planned to guide fluid treatment decisions during the initial resuscitation phase in patients with sepsis with real-time monitoring of ECW/TBW variables.

This study had several limitations. First, MAP was used as a control variable for fluid administration and vasopressor due to the practical limitation of incessant preload responsiveness test and unfeasibility of passive leg raising in progressive septic shock model [39, 40]. However, after the initial loading fluid (30 ml/kg), additional fluid loading was attempted based on the response of MAP. Second, inter-subject variability in terms of inherent cardiovascular function may exist since the number of subjects was 12. To minimize the bias possible, only healthy male pigs from a single farm and a single type of bacteria (ESBL-producing *E. coli*) were used. Therefore, extrapolation to female pigs or humans with other pathogens should be approached with caution when interpreting these results. Third, the absolute values of ECW, ICW, and TBW were not provided in this study. Because the equations used to calculate body water compartments are validated in healthy, euvolemic human adults, these equations cannot be applied to porcine subjects. Therefore, to evaluate the trend of body water distribution change, the ratio of body water compartments rather than absolute values, is presented. Fourth, only one type of BIA equipment was used (InBody M20). The measured and calculated values may differ if other bioimpedance equipment are used. Fifth, although the experiments were aimed at MAP > 65 mmHg, massive hydration resulting in fluid balance > 5000 mL is far from the usual clinical setting. Further clinical studies are needed to validate the feasibility of BIA in assessing body water distribution during fluid resuscitation in patients with sepsis.

In conclusion, we demonstrated the feasibility of BIA during fluid treatment for sepsis. Among the variables, the trend of ECW/TBW increased and the phase angle decreased during the fluid treatment, which was accentuated in non-survivors with increasing cumulative fluid balance. The correlations between the hourly fluid balance and ΔR_0_, ΔECW/TBW, and Δphase angle further emphasize the result. This suggests the potential of BIA to measure fluid balance in real-time and provide body water status to clinicians during fluid therapy in patients with sepsis. Additional studies including clinical trials should be performed to suggest criteria for discontinuing fluid administration or increasing the vasopressor dose instead of fluid treatment.

## Supplementary Information

**Additional file 1: Figure S1**. Trends of P/F ratio, urine output, creatinine, platelet, total bilirubin and albumin levels of 12 pigs in the ESBL-producing *E. coli*-induced porcine bacteremia model. Data are presented as the means and standard error of the mean. P/F ratio: ratio of partial pressure of oxygen to the fraction of inspired oxygen.

## Data Availability

The datasets used and/or analyzed during the current study are available from the corresponding author on reasonable request.

## Abbreviations

ECW: Extracellular water
TBW: Total body water
ESBL: Extended-spectrum β-lactamase
*E. coli*: *Escherichia coli*
ICU: Intensive care units
BIA: Bioelectrical impedance analysis
PCWP: Pulmonary capillary wedge pressure
ICW: Intracellular water
MAP: Mean arterial pressure
HR: Heart rate
PAP: Pulmonary arterial pressure
CVP: Central venous pressure
CO: Cardiac output
SV: Stroke volume
SVR: Systemic vascular resistance
ABGA: Arterial blood gas analysis
P/F ratio: Ratio of partial pressure of oxygen to the fraction of inspired oxygen
SOFA: Sequential Organ Failure Assessment
CNS: Central nervous system
RM ANOVA: Analysis of variance for repeated measures
BPM: Beats per minute
VIS: Vasopressor-inotrope score
BIVA: Bioelectrical impedance vector analysis
