# Supplementary material for: Feasibility study using longitudinal bioelectrical impedance analysis to evaluate body water status during fluid resuscitation in a swine sepsis model
Source: Intensive Care Med Exp. 2022 Dec 6;10:51. doi: 10.1186/s40635-022-00480-5 (PMC9727062; doi:10.1186/s40635-022-00480-5)
Supplement: Supplementary file 1 — Additional file 1: Figure S1. Trends of P/F ratio, urine output, creatinine, platelet, total bilirubin and albumin levels of 12 pigs in the ESBL-producing E. coli-induced porcine bacteremia model. Data are presented as the means and standard error of the mean. P/F ratio: ratio of partial pressure of oxygen to the fraction of inspired oxygen. [file 40635_2022_480_MOESM1_ESM.pdf]

Online Data Supplement for

Feasibility study using longitudinal bioelectrical impedance analysis to evaluate body water status during fluid resuscitation in a swine sepsis model

Hwain Jeong, Inwon Park, Jae Hyuk Lee*, Dongsung Kim, Sumin Baek, Seonghye Kim, You Hwan Jo

* Corresponding authors. E-mail: hyukmd@gmail.com (J.H.L)

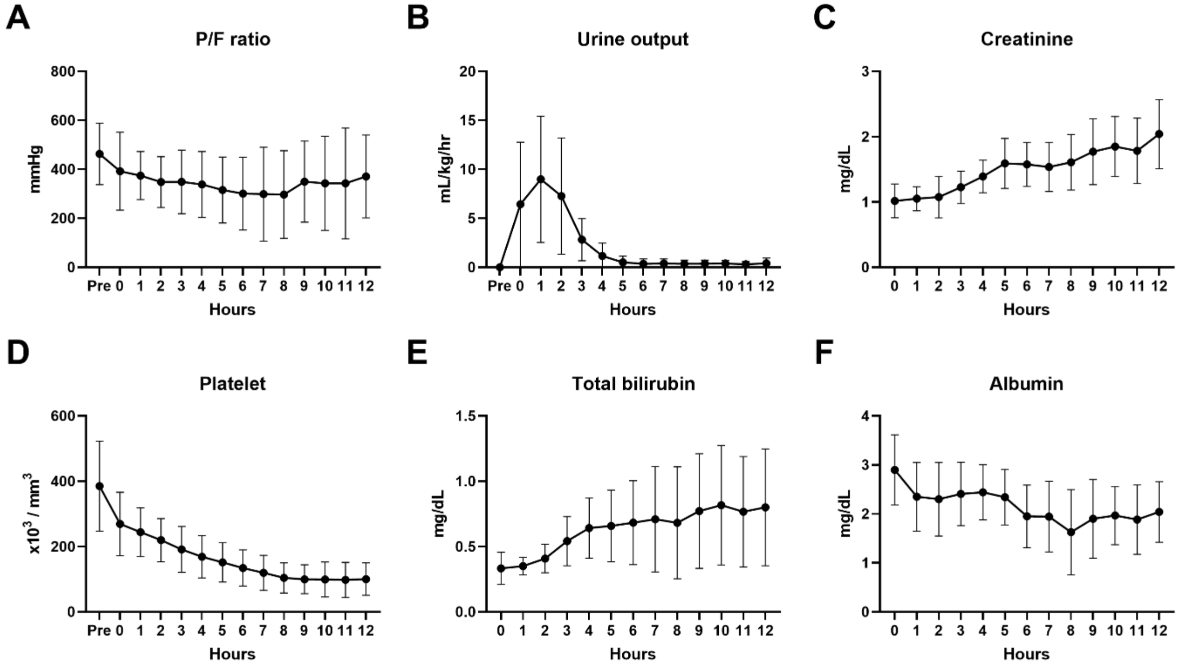

Supplementary Figure 1. Trends of P/F ratio, urine output, creatinine, platelet, total bilirubin and albumin levels of 12 pigs in the ESBL-producing *E. coli*-induced porcine bacteremia model. Data are presented as the means and standard error of the mean. P/F ratio: ratio of partial pressure of oxygen to the fraction of inspired oxygen.
